# Docking Studies of Pakistani HCV NS3 Helicase: A Possible Antiviral Drug Target

**DOI:** 10.1371/journal.pone.0106339

**Published:** 2014-09-04

**Authors:** Kaneez Fatima, Shilu Mathew, Mohd Suhail, Ashraf Ali, Ghazi Damanhouri, Esam Azhar, Ishtiaq Qadri

**Affiliations:** 1 IQ Institute of Infection and Immunity, Lahore, Punjab, Pakistan; 2 Center of Excellence in Genomic Medicine Research, King Abdul Aziz University, Jeddah, Saudi Arabia; 3 King Fahd Medical Research Center, King Abdul Aziz University, Jeddah, Saudi Arabia; University of Colorado, Denver, United States of America

## Abstract

The nonstructural protein 3 (NS3) of hepatitis C virus (HCV) helicase is believed to be essential for viral replication and has become an attractive target for the development of antiviral drugs. The study of helicase is useful for elucidating its involvement in positive sense single-stranded RNA virus replication and to serve as templates for the design of novel antiviral drugs. In recent years, several models have been proposed on the conformational change leading to protein movement and RNA unwinding. Some compounds have been recently reported to inhibit the helicase and these include small molecules, RNA aptamers and antibodies. The current study is designed to help gain insights for the consideration of potential inhibitors for Pakistani HCV NS3 helicase protein. We have cloned, expressed and purified HCV NS3 helicase from Pakistani HCV serum samples and determined its 3D structure and employed it further in computational docking analysis to identify inhibitors against HCV genotype 3a (GT3a),including six antiviral key molecules such as quercetin, beta-carotene, resveratrol, catechins, lycopene and lutein. The conformation obtained after docking showed good hydrogen bond (HBond) interactions with best docking energy for quercetin and catechins followed by resveratrol and lutein. These anti-helicase key molecules will offer an alternative attraction to target the viral helicase, due to the current limitation with the interferon resistance treatment and presences of high rate of resistance in anti-protease inhibitor classes.

## Introduction

Hepatitis C virus (HCV) is one of the major causative agent of chronic hepatitis which leads to liver cirrhosis, hepato cellular carcinoma, and liver failure and the most significant cause for liver transplantation [1], [2]. It is estimated that about 3% of the world's population (∼180 million people) are affected with HCV [3] and 10 million people are believed to be infected by HCV alone in Pakistan [4]. HCV RNA genome encodes a single open reading frame that is translated into 3,000 amino acids (AA) poly protein and cleaved into 10 mature proteins. HCV genome translated into 4 structural (Core, E1 E2 and p7), and 6 important nonstructural (NS) proteins: NS2, NS3, NS4A, NS4B, NS5A, and NS5B [5], which coordinate the intracellular processes of the viral life cycle. Among the NS proteins, NS3 is a multifunctional protein (1–631 AA) with serine protease activity at the N-terminal (1–180 AA) and a nucleoside-triphosphatase (NTPase) dependent RNA helicase activity (NS3 NTPase/helicase) at the C-terminal (181–631 AA) [6].

Among all HCV proteins, NS3/NS4A serine protease and helicase are effective drug targets to develop anti-HCV agents [7]. The basic role NS3/NS4A is to cleave virus at different functional points as well as involved in viral replication. NS3 RNA helicase affects two different steps in the virus life cycle: (a) RNA replication step of virion in which NS3 is required to unwind the double-stranded RNA intermediate during RNA-dependent replication, that enables the movement of HCV NS5B polymerase [8], (b) NS3 assists in virus assembly and can also act as a scaffold for interaction with viral or cellular cofactors [9], [10]. The crystal structure of HCV helicase shows that it consists of motifs I, Ia, II, III, IV, V, and VI, which are highly conserved. These motifs are located in the ATP binding cleft, and some project residues located at the nucleic acid binding site.

Recently two NS3 protease inhibitors have been approved as a standard care for HCV GT1 affected patients by providing treatment with triple therapy (Peglated-Interferon - α, ribavirin and boceprevir or telaprevir [11] that are available in the market under the brand name Victrelis for boceprevir or Incivek and Incivo for telaprevir. In patients with GT1chronic HCV infection, the treatments with telaprevir/boceprevir based triple therapy are standard-of-care. However, more efficacious direct-acting antivirals (DAA) (Interferon (IFN)-based new DAAs) are available and interferon-free (IFN-free) regimens are imminent in near future. Imminent treatments for individuals infected by HCV will likely involve combinations of compounds that inhibit multiple viral targets. HCV helicase is an attractive target with no available drug candidates in clinical trials. Herein we describe an integrated strategy for identifying fragment inhibitors using computational techniques. Due to increase in HCV infection cases and lack of effective therapies, there is a need to develop specific compounds that can target the HCV [12]. Therefore, this study was planned to molecularly characterize the Pakistani HCV helicase protein. We cloned, purified HCV helicase, determined its 3D structure and docked with different available inhibitors chosen from the family of bioflavonoids. The flavonoids are significant source for developing new antiviral agents. Using computational docking study, we determined active inhibitors against genotype 3a (GT3a) NS3 helicase strain to pave a way to treat HCV patients in Pakistan.

## Methodology and principal findings

### 2.1 Cloning and expression of Pakistani HCV helicase

The HCV helicase in this study was derived from our reported HCV NS3/NS4A expression clone (Accession no. FJ839678) [13], obtained from the Pakistani HCV serum samples collected from the Holy Family Hospital, Rawalpindi and were a kind gift from Dr. Omar Ahmad and Dr. Zahid. The NS3/NS4A of genotype 3a (GT3a) was PCR amplified by using site specific primers following the cloning into pET28 (a) expression vector. In this construct, the last six amino acid residues “DLEVTT” from the 3′ end of helicase have been deleted to get the high yield of helicase expression and purification. The set of primers were designed for cloning into pET28 (a) vector by using site specific restriction endonucleases: Forward primer (NS3hF 5′-AAA*GCTAGC*TCAACTCCTCCTGCTGTTCCACAG-3′ the *NheI* site is underlined) and reverse primer (NS3hR-CCC*GCGGCCGC*TTAAGCTGACATGCTTGCCATGATGTA-3′ the *NotI* site is underlined). The pET-NS3-helicase construct was carrying His_6_ tag at C-terminus of the protein to facilitate purification, and was expressed in *Escherichia coli*, Rosetta DE3.

**Figure 1:**
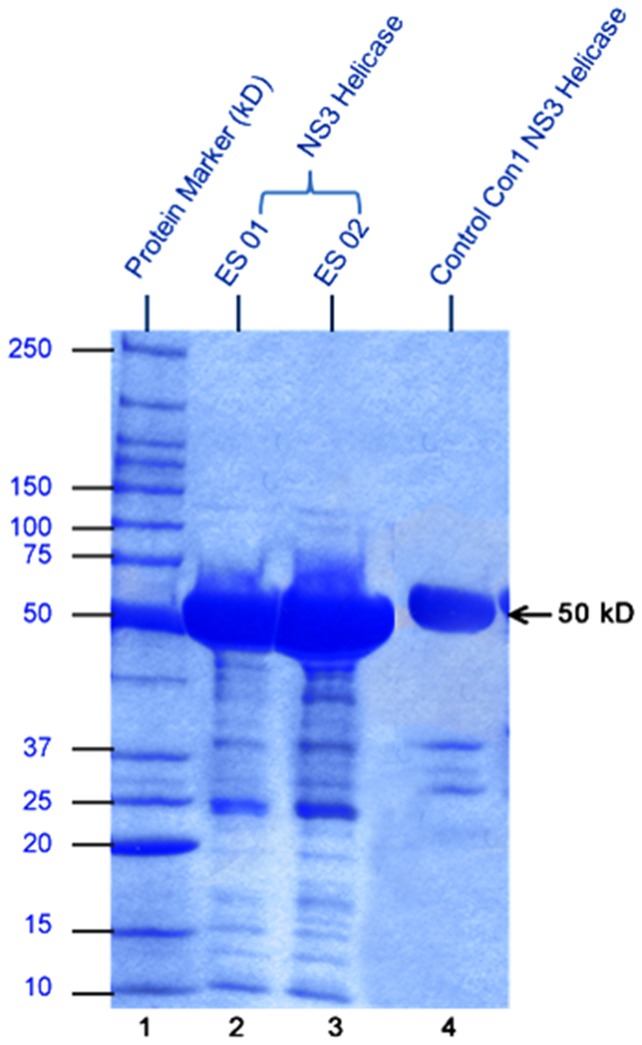
4–20% gradient SDS-PAGE for expression of HCV NS3 helicase protein GT3a. Lane 1, Molecular Mass Marker; Lane 2–3, NS3 helicase GT3a expression strain 01 and strain 02 respectively; Lane 4, Control Con1 GT1bhelicase.

The expression of this construct was tested at a small scale by using the method optimized in Dr. Charles M. Rice lab [14]. A discrete protein band of 50 kDa was identified on sodium dodecyl sulfate-polyacrylamide gel electrophoresis (SDS-PAGE) in parallel to the control NS3h ‘Con1-1b genotype’ shown in Figure 1. After assessment of protein expression level at lab scale, the protein was expressed at large scale for purification. The expression was induced by addition of 1.0 mM IPTG at 30°C for4 h. The bacterial cell culture was harvested by centrifugation at 3000 rpm for 20 min and 16 g pellet was re-suspended in re-suspension buffer(5 M NaCl, 2 mM β-imidazole, 10% IGEPAL, 100 mM PMSF, 14 M β-ME, 10 mg/ml DNase I (Fermentas, Cat# EN0523), 5 mg/ml RNase A (Fermentas, Cat #EN0531), and 50 mg/ml lysozyme). The cells were lysed by freezing and thawing, followed by sonication using sonifier for 90 s/pulse at level one for the first pulse following the 4–5 pulses for 30 seconds.

The NS3h protein with His6 tag was bound to the Nickel Sepharose pre-equilibrated with there-suspension buffer and washed with the washing buffer (500 mM NaCl, 20 mM imadizole, 20 mM Tris–HCl (pH 8.5) and 1 mM β-ME). The bound protein was eluted with elution buffer (500 mM NaCl, 240 mM imidazole, 20 mM Tris pH 8.5 and 1 mM β-ME). The eluted protein was subjected to Sodium dodecylsulphate-polyacrylamide gel electrophoresis (SDS-PAGE) and stained with Coomassie Brilliant Blue R-250 (CBBR-250). The fractions were pooled for the following purification steps.

### 2.2 Purification of NS3 helicase by *Fast protein liquid chromatography* (FPLC)

**Figure 2:**
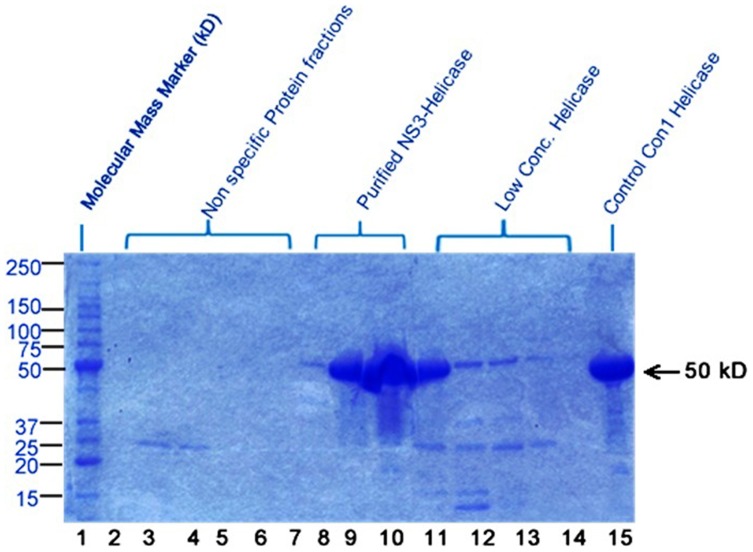
20% SDS Gel showing purified protein by ion exchange chromatography. Lane 2–7, Different fractions with non-specific proteins; Lane 8–10, NS3 helicase GT3a purified and separated fractions after FPLC; Lane 11 & 14, NS3 helicase with low concentration and non-specific proteins; Lane 15, Control Con1 GT1b helicase.

Affinity Chromatography was performed by using Nickel-Nitrilotriacetic acid (Ni-NTA kit). After semi purification from NI-NTA kit, the respective protein was further purified by FPLC using the Ion Exchange Column Chromatography method optimized in Rice lab [14]. The samples were prepared, filtered and collected in a syringe with the injection needle and run for overnight. The purified protein was collected and the concentration was measured by Bradford assay. The desired eluted protein was found to be present with traces of other non-specific protein (Figure 2). Approximately 14 mg/ml of purified (>95%) protein was obtained following the purification. The purified protein was stored at −20°C for further study.

### 2.3 Structure of Pakistani HCV NS3 helicase

The first crystal structure of HCV NS3 was first purified by Yao et al in 1999 [11], [15]. The structure of the HCV NS3 helicase was determined by multiple isomorphous replacement (MIR) combined with anomalous scattering (MIRAS). The protein consists of three domains separated by a series of clefts. The reported structures of HCV helicase shared similar global conformation consisting of three domains and can be viewed as a Y-shaped molecule, the most N-terminal domain (domain 1) and the middle domain (domain 2) are above the C-terminal domain (domain 3) [15]. The sequence length of GT3a NS3/NS4A is 705 AA, with a chain length 1–651 AA consists of protein NS3 and 651–705 AA positions holds protein NS4A [13].

### 2.4 Pairwise and phylogenetic alignment

The target FASTA sequence of the GT3a NS3/NS4(Accession no: FJ839678) having 705 AA residues and the template HCV domain of genotype 1a (GT1a) having 3011 AA residues(Accession no: P27958) were aligned by pair wise alignment with CLC drug discovery workbench to define the identities and the conserved region [16]. The pairwise alignment parameters include gap open penalty 10, gap extension penalty 0.1, by using BLOSUM weight matrix.

### 2.5 Pfam domain search

The Pfam domain were predicted for GT3a NS3/NS4Awith the score and e-value from 100 most common domains predicted by CLC Drug Discovery Workbench 1.0.2 [16] by comparison from full length protein model.

### 2.6 Generating and validating modeled protein

Comparative modeling was done by using Modeler 9.2 [17]–[20] by assembling the determined rigid structure to model the Pakistani HCV GT3a NS3 helicase from the FASTA sequence determined as target [13]. The input protein sequence of GT3a retrieved from UniProtKB/Swissprot (Accession No. C1KIT2) was screened as target from structural databases [13]. Based on the protein BLAST, the UniProtKB: C1KIT2 shared highest sequence identity (83%) and the total query coverage (89%)with X-Ray structure of HCV isolated from genotype 1 (PDB ID: 1CU1) which has been elucidated at 2.50 A^0^ [11]. The HCV NS3 helicase domain of genotype 1a (GT1a) strain, is 476 AA residues chain length (UniProtKB: P27958) was used as a template to model the Pakistani GT3a NS3 helicase (UniProtKB ID: C1KIT2) [13], [21]. Three dimensional structure of HCV GT3a NS3 helicase was built based on the target-template alignment using a subset of atomic positions from the template structure as guiding factor. Ten models were built using MODELLER 9.12 [20] and the modeled protein residues ranged from 210 to 651AA residues of target based upon the similarity match with the template. Further, energy minimization of the modeled protein was done by using ModRefiner. The NS3 model was refined geometrically in order to decrease the side chain steric clashes by using online tool ModRefiner [19], [22]. This algorithm refines protein structure for atomic level with high resolution [22]. It follows two-step procedure for constructing full-atom model with initial C-alpha trace. The initial step builds the backbone for the available C-alpha and conducts energy minimization to improve the quality. Second step involves addition of side chain atoms from a rotamer library and conducts energy minimization to both side chains and backbone conformations [22]. The stereo chemical quality of the helicase modeled structure was analyzed by PROCHECK algorithm that evaluates residue by residue as well as overall geometrical structure. The volume of atoms in the modeled macromolecule, compatibility of each amino acid sequence, statistics of non-bonded interactions and extensive screening for stereo chemical parameters for the residues were analyzed by PROVE, VERIFY_3D, ERRAT and WHAT_CHECK from Structural Analysis and Verification Server (SAVES) [23], [24]. The 3D model was visualized by UCSF Chimera package [25]. SuperPose Version 1.0 server was performed to calculate 3D modeled helicase superimposed with template protein by using modified quaternion approach. This server generates Root mean square deviation (RMSD) statistics, PDB coordinates, interactive images of both sequence and structural alignment of the superimposed structures [26].

### 2.7 HCV NS3 helicase inhibitors

**Figure 3:**
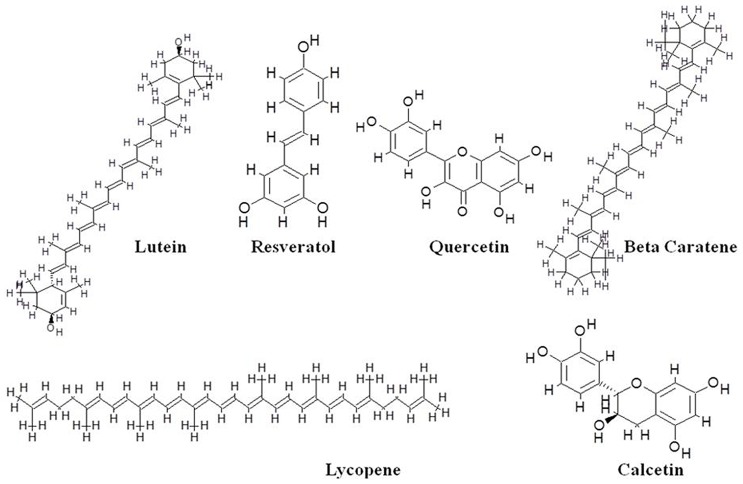
Two-dimensional structure of antiviral agents generated against HCV GT3a NS3 helicase.

Though the development of the helicase inhibitor has been far slower than other HCV drug targets [27], [28]. There are only a few classes of helicase inhibitors that have been reported to slow HCV RNA replication in cell culture system of viral replication. HCV helicase inhibitors reported to act as antiviral include nucleoside mimics [29] triphenylmethanes [30], acridones [31], [32], amidinoanthracyclines [33], tropolones [34], symmetrical benzimidazoles [35]–[37] and primuline derivatives [38]. A very few studies have been reported targeting NS3 helicase by using flavonoid. Bioflavonoids are the secondary metabolites occurring naturally from plants; possess a wide spectrum of antiviral properties including the inhibition of viral replication, translation and other steps of infection [39]. Bioflavonoids such as quercetin, beta-carotene, resveratrol, catechins, lycopene and lutein were chosen to study the interaction with the modeled HCV helicase. These effective bioactive compounds have been reported to interfere with many disease associated biochemical processes *in vitro* [40]. Quercetin is reported in inhibiting initial stage of viral replication [41]. Previous studies have demonstrated that the bioflavonoid quercetin blocks HCV proliferation by inhibiting through internal ribosomal entry site (IRES) - mediated translation of the HCV viral genome [40]. Flavonoids are well known for their significant antiviral inhibition activity, associated with no cytotoxicity [40]. Catechins being a polyphenolic compound from green tea were best evaluated for altering physical property of influenza viral membrane and voted for direct virucidal activity [42], [43]. Studies have reported beta-carotene: a major precursor for vitamin A if maintained through proper supplementation is beneficial to boost the immune against viral and tumor surveillance [44]. In recent years, deep research is carried out for resveratrol produced by certain plants on various stimuli, is well known for its antiviral activity through various molecular pathways [45]. Though numerous epidemiological studies have pointed that consuming abundant carotenoids calm down the risk of various cancer, the protective mechanism of carotenoids based on their anti-oxidant capability, makes it a promising chemo preventive molecule that can be targeted for antiviral character. Lutein and lycopene, abundant in fruits and vegetables also possess strong anti-oxidant property [46]. Lutein inhibition activity against HBV full-length promoter was exposed *in vitro*, through data which introduced lutein exerts antivirus effects via inhibition of HBV transcription cycle [47]. Therefore, the listed flavonoids are well known to stand high for their anti-oxidant property, which led us to recruit them as inhibitors [42]. The chemical structures of selected compounds are represented in Figure 3. The 2D structures were downloaded from PUBCHEM in SDF format which were converted to PDB by using NCI Online SMILES Translator [48].

### 2.8 *In silico* docking

Further refinement of the retrieved targeted compounds, were docked with the modeled GT3a NS3 helicase by using CLC Drug Discovery Workbench software package [16]. Basic set ups like protein preparation, ligand preparation, detecting cavities, receptor grid generation and targeted ligand docking were performed. Docking wizard was customized by using default MolDock optimizer algorithm with 200 numbers of runs. The docking parameters include population size 50, maximum iterations 2000, scaling factor 0.50, crossover rate 0.90 and RMSD thresholds for similar cluster poses were set as 1.00. CLC Drug Discovery Workbench uses a standard precision mode to determine the favorable binding poses, which detects various flexible ligand conformations while holding protein as rigid structure during docking. Quercetin, beta-carotene, resveratrol, catechins, lycopene and lutein, were docked into active site recognized in the macromolecule cavity. For comparative analysis, ten active compounds were selected as they were reported in inhibiting the HCV NS3 helicase activity. Maximum of 10 poses for each conformations were generated by using default parameter of CLC Drug Discovery Workbench. Docking studies were carried out to predict the binding affinities based on scoring functions. On the basis of hydrogen interaction and docking score, the best ranked compounds were selected and their binding residues were observed by using CLC drug discovery visualization tool [49].

## Result and Discussion

### 3.1 Structural alignment and domain analysis

**Figure 4:**
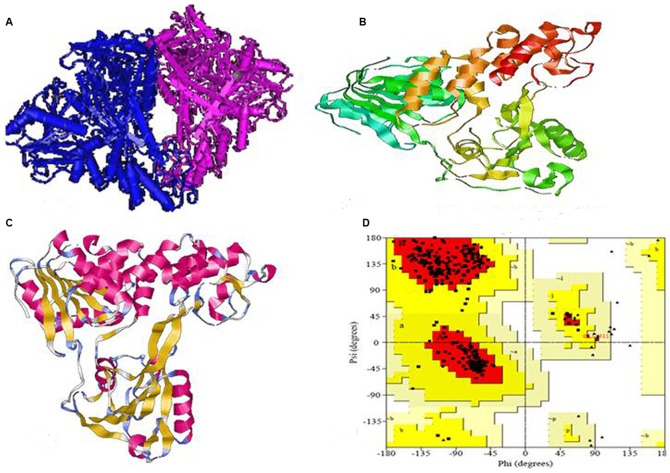
Structures of HCV NS3 Helicase (A) Ribbon form of 1CU1 protein, (domain A; dark blue and domain B; purple) (B) crystal structure of helicase 1A1V. (C) Superimposed macromolecule of 1CU1 (yellow) and 1A1V (pink). (D) Ramachandran contour plot of helicase NS3.

**Figure 5:**
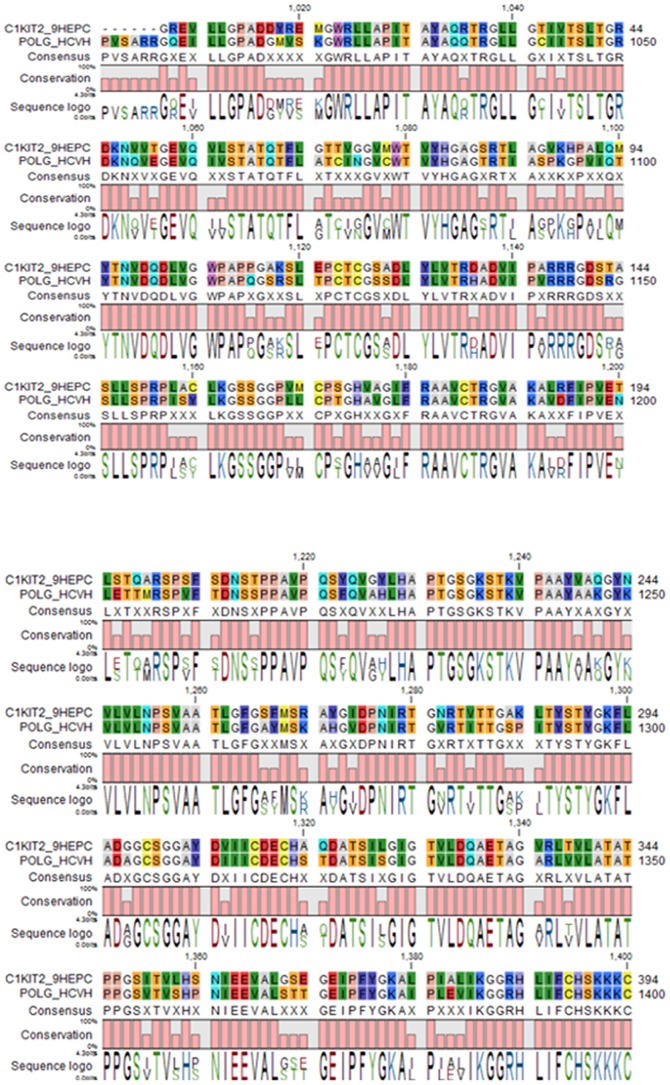
Pairwise alignment between HCV GT1a and GT3a NS3/NS4A by using CLC drug discovery workbench. Amino acid pairwise alignment between target (705 AA) and template (3011 AA) showed 80.28% similar for conserved regions.

**Figure 6:**
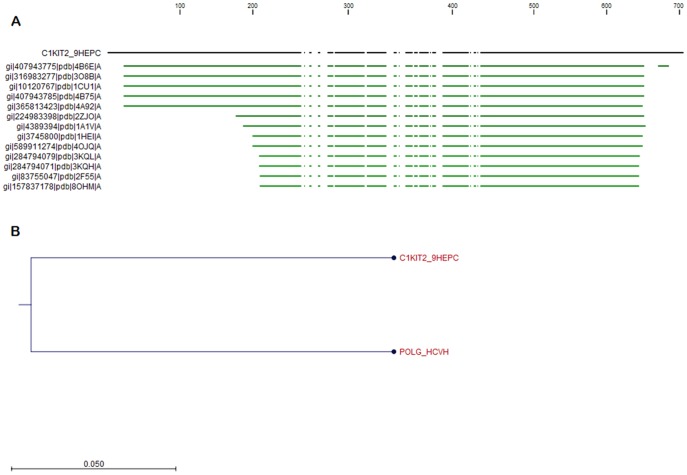
BLAST alignment and phylogenetic tree of GT3a NS3 helicase. (A) The green line indicates the template sequence with maximum similarity with the target sequence (B) The amino acid substitution rate between GT3a NS3/NS4A and HCV GT1a is 0.05%.

The BLASTp search program revealed several sequence homologous to helicase polyprotein (UniProtKB id: C1KIT2) but HCV helicase domain (PDB code 1A1V, represented in Figure 4A) was chosen as the best template for modeling GT3a helicase. The multiple sequence alignment score between the target (705 AA) and template (3011 AA) showed 80.28% pairwise similarity by using CLC drug discovery workbench shown in Figure 5. The AA substitution rate between GT3a NS3/NS4A and HCV genotype 1a (GT1a) is 0.05% also shown in Figure 6. The Pfam domain predicted for GT3a NS3/NS4Ais presented in Table 1 with the score and e-value.

**Table 1:** PFAM domain search table.

Start	End	Domain	Accession	Score	E-value
15	190	Trypsin	PF00089	−78.10	0.54
62	432	Major Facilitator Superfamily	PF07690	−100.10	0.85
98	434	Aminotransferase class I and II	PF00155	−165.10	0.92
122	137	ATPase family associated with various cellular activities (AAA)	PF00004	−0.30	0.23
159	173	Trypsin	PF00089	−1.10	0.35
209	352	DEAD/DEAH box helicase	PF00270	−31.80	2.10E-4
222	231	DEAD/DEAH box helicase	PF00270	−1.50	0.63
400	489	Helicase conserved C-terminal domain	PF00271	2.30	5.70E-4
480	489	Helicase conserved C-terminal domain	PF00271	−0.70	0.92

Therefore we generated a 3D macromolecule from the target GT3a NS3from 210 to 651AAas they were aligned and modeled from chain A containing 476 AA sequence length (1A1V) of helicase of GT1a by homology modeling procedure. The template 3D structure of NS3 helicase domain of 1A1V with its chain A and B is represented in ribbon structure in Figure 4A. The modeled GT3a NS3helicase structure and its Ramachandran plot containing the phi and psi values are shown in Figure 4B and 4D with 93.7% residues in the most favored regions, followed by 6.0% in the additionally allowed region and 0.00% in disallowed region. The RMSD value of the superimposed modeled and template structure is 1.27A^0^ (presented in Figure 4C). The calculated RMSD value is between the main-chain atom of model and template, which indicates close homology, ensuring good reliability of the model helicase [22].

### 3.2 Binding Interaction of HCV NS3 helicase

**Figure 7:**
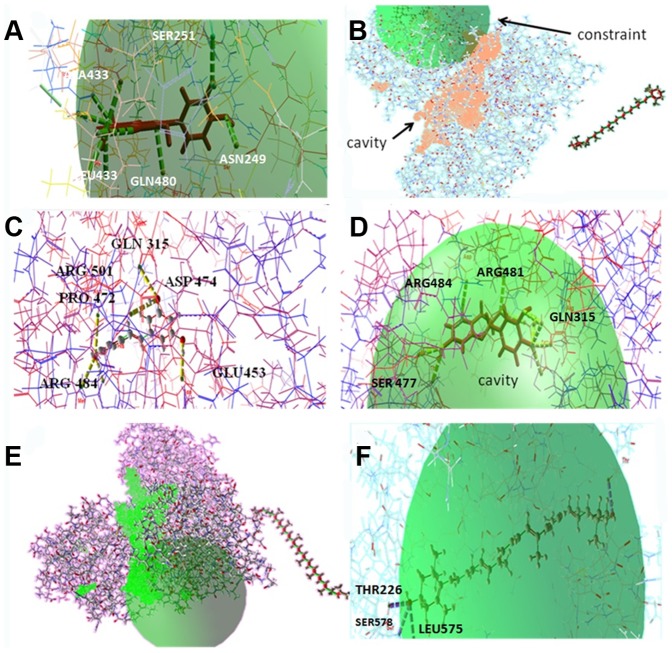
Docking conformations and binding pockets of HCVGT3a NS3 helicase with different inhibitors. (A) Three-dimensional representation of quercetin with target macromolecule and its hit residues (B) Beta-carotene represents no interactions in its constraints with the modeled structure. The Hbond formation is shown in stick mode (green). (C) Best pose of the compound resveratrol forming seven HBonds with three-dimensional structure of helicase at its binding sites GLN 315, GLU 453, ARG 484, PRO 472, ASP 474, ARG 501 (D) Three-dimensional representation of catechins and target molecule with eleven HBonds network. The Hbond formation is shown in stick mode (yellow) and the constraint is shown in green color. (E) Zero interactions between lycopene and modeled GT3a NS3 helicase (F) binding of lutein at THR 226, LEU 575 and SER 578 residues. Lutein forming three HBonds with high HBond energy −2.5461 kcal·mol^−1^.

**Figure 8:**
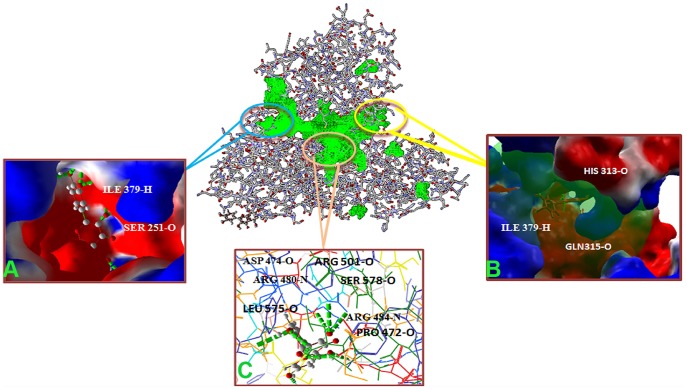
Per residue interaction of HCV GT3a NS3 helicase at specific domains (A) Helicase ATP-binding (B) NS3-binding (C) DECH box. The modeled GT3a NS3 helicase protein is shown in cartoon and colored in gray. Binding cavities and HBonds are represented in green color.

The docking studies of GT3a NS3helicase were carried out to define the binding pockets, inhibitors interactions, and their specificity and energy requirement. Our *in silico* docking study using MolDock scores observed for the modeled GT3a NS3 helicase and the six ligands are summarized in Table 2. Ten different conformations with best pose between protein ligand interactions were generated based on HBond distances. The best-ranked docked conformation was considered for binding affinity study by measuring HBond interactions. Depending on two parameters, the potential inhibitors were chosen based on 1) details of HBonds of best-ranked pose and 2) binding energy predicted between docked flavonoids and the protein. In our analysis, the compounds catechins and quercetin fitted well in active pockets of GT3a NS3 modeled helicase presented in Figure 7, denoting minimum docking energy values and formed maximum number of HBonds compared with other flavonoid. The best pose of interaction for quercetin showed fourteen HBonds interactions with its active site residues such as SER 251, ASN 249, GLU 311, GLN 480, ALA 433, LEU 434, ASP 432, ASP 425, ARG 484, GLN 480, ILE 379 and ASP 474. In the docked complex, quercetin and catechins revealed H Bonds formation with similar active site residues ARG 484 and ASP 432, which denote region for NS3 binding motifs. Catechins interacted with GLN 315, GLU 453, SER 477, ARG 481, HIS 313 and VAL 426 with minimum docking energy (Figure 7C). The HBonds formed between each flavonoid compound and the modeled NS3 helicase with its docking energy, MolDock score, HBond energy and number of residue interaction with labels are denoted in Table 2. The HBond energy values for both quercetin and catechins were −9.4 kcal·mol^−1^and −7.2 kcal·mol^−1^. The other two inhibitors like resveratrol displayed seven HBonds interaction with HBond energy and docking energy as −7.2 kcal·mol^−1^ and−95.41 kcal·mol^−1^, whereas lutein exhibited only three HBonds interaction with THR 226, LEU 575 and SER 578 with high HBond energy −2.54 kcal·mol^−1^. Beta-carotene and lycopene showed no interactions with the active residues of the modeled helicase might be due to their long chain of carbon atoms and presence of isoprene units as they both are members of tetraterpenes, synthesized biochemically. The docking interactions and the number of HBonds formed between each bioflavonoid are shown in (Video S1). The MolDock score increased with respect to the number of hydrocarbon atoms in the targeted compounds. The number of torsions angles chosen for ligand to fit into the binding pockets of active site residues also showed higher for isoprenoid compounds. Since the docking result suggests that the CLC drug discovery software reproduced appropriate conformations of selected flavonoid inside the binding pockets of NS3 helicase active site suggesting a competitive inhibition of the helicase. The ATP binding position (1217–1369 AA), NS3 binding motifs site (1679–1690 AA) and DECH box (1316–1319 AA) of the template 1A1V was compared to determine the frequent binding interaction at the conserved sites of the respective regions of the modeled helicase (Figure 8). The binding modes depict ILE 379 and SER 251 reside DECH box region. Frequent binding was spotted at NS3 binding site by quercetin, resveratrol and lutein. Only quercetin and catechins showed HBonds interaction at DECH box. The interaction of specific residues with the potential regions was confirmed from GT1a through sequence annotation along with feature and region description of residue positions. The confirmation obtained after docking showed good energy binding and docking energy for quercetin, catechins, resveratrol and lutein. Quercetin and catechins demonstrated stronger *in silico* inhibition of the infectious virion helicase compared to resveratrol. Lutein demonstrates mild interaction compared to other bioflavonoid. Therefore the flavonoid compounds that presented high HBond interaction and closest binding energy values to modeled GT3a NS3helicase were considered to be the best results. This order of specificity indicates that the flavonoids catechins and quercetin followed by resveratrol and lutein showed good inhibition activity towards the modeledGT3a NS3 helicase.

**Table 2:** Targeted residues in interaction and their docking score.

Name of the Derivative	MolDock Score	RMSD	Hbond	Interactions	Docking Score	Name Of the bond	Number of hydrogen bonds
Quercetin	−86.6863	37.518	−9.4567	14	−94.8608	UNK-O UNK-C	SER 251-O ASN 249-O GLU 311-O GLN 480-O ALA 433-O LEU 434-O ASP432-O ASP 425-O ARG 484-N GLN 480-O ILE 379-H ASP 474-O
Beta-Carotene	−133.204	57.4964	−149.249	0	−130.601	-	-
Resveratol	−89.8886	37.1295	−7.3495	7	−95.4149	UNK-O UNK-C	GLU 453-O ARG 484-N PRO 472-O ASP 474-O ARG 501-O
Catechins	−88.3722	41.8008	−7.2845	11	−99.5567	UNK-O UNK-C	GLN315-O GLU 453-O SER 477-O ARG 481-N ARG 484-N ASP 432-O HIS 313-O VAL 426-O
Lycopene	−113.582	36.6534	−136.284	0	−109.635	-	-
Lutein	−120.047	42.4308	−2.5461	3	−119.747	UNK-O	THR 226-O LEU 575-O SER 578-O

Very few *in silico* studies have shown different inhibitors targeted against helicase and their binding specificities. A list of *in silico* study carried out against viral helicase as targets from HCV is shown in Table 3. Though HCV comes under the family of flaviviridae, its helicase is considered as template for comparative study with other vital viruses. Use of natural photochemical from plants is a promising therapy, reported by a study were medicinal plant *Amelanchier alnifolia* and its component quercetin followed by 3-galactoside and 3-glucoside showed interactions with protease and helicase respectively [50]. Drug therapy target against NS3 resistant variants R155K and V36M was also reported with impact on conformation of the beta-barrel domain of the viral protein. This domain is involved in substrate binding and in active site binding pocket [51]. Therefore, our docked results provide potential information for new inhibitors analogues targeted towards helicase, to fight against HCV through drug-flavonoid pharmacokinetic interactions.

**Table 3:** Inhibitors of helicase available until now and their binding specificities.

Drugs	Residues	References
Ivermectin	T408, D409	[52]
Quercetin,3-galactoside, 3- glucoside	ALA157, HIS528	[53]
Mercapto compounds	CYS431,ARG393,ARG481	[54]
VX-950,tri-andtetra peptides, hexapeptides	Q526A, H528A, H528S	[55]
Dihydropyrols, Phenylalanine analogs, Thiophenes, Benzofurans, Phenylpropanyl Benzamides, Benzimidazoles, Indoles	ASP318,SER556,ASN291, SER 367, SER476, TYR477	[56]

### 3.3 Comparative docking study from bioassay hit compounds

**Figure 9:**
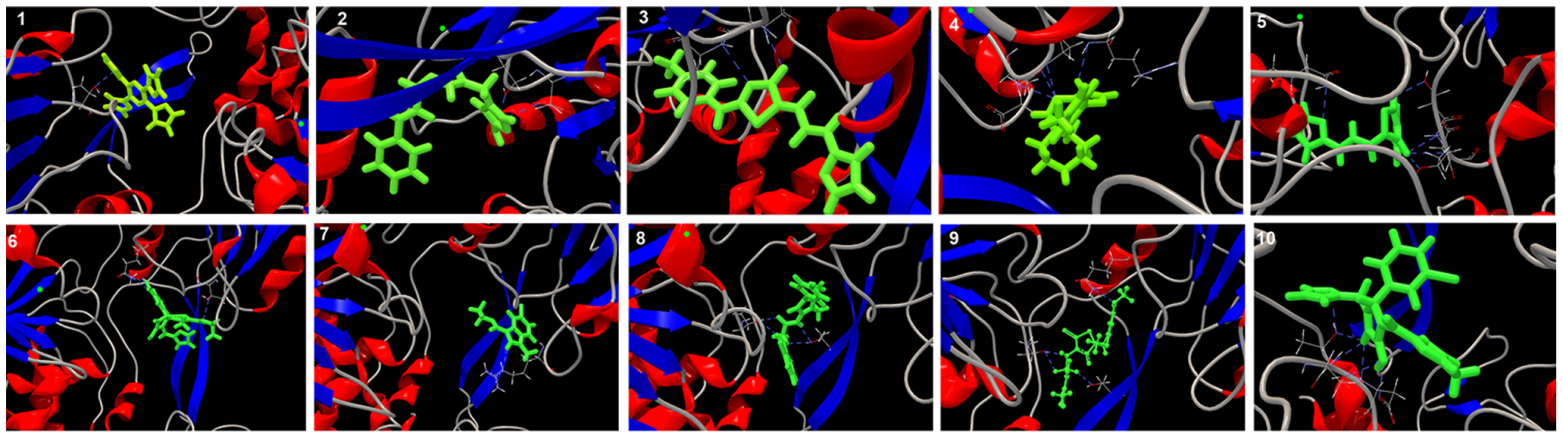
Comparing the docking interaction with ten hit compounds with HCV GT3a NS3 helicase such as 1) SID 17513061 2) SID 3716320 3) SID 17513201 4) SID 17401675 5) SID 49666882 6) SID 24818609 7) SID 24827353 8) SID 49732586 9) SID 4257236 10) SID 49817864 obtained from PubChem Bioassay.

A comparative study of the interaction of active ten compounds from PubChem bioassay were considered for docking with the modeled GT3a NS3 has been presented in Figure 9 exploiting the interaction of the bound drug. Molecular docking studies were performed to provide further insight into various other drug interactions which were identified as inhibitors of the hepatitis C virus NS3 through fluorescence-based primary biochemical high throughput screening. The lowest score was selected corresponded to the best docking pose as well as the number of HBonds formed. The docked conformations of the ten hit compounds in the GT3a NS3complex are shown in Figure 9. Table 4 denotes the interaction energy and the HBonds formed with the modeled residues. Thus, it has been observed that the number of residues involved in binding is higher is maximum for bioflavonoids catechins and quercetin whereas in the case of ten hit compounds selected the number of residues perturbed was found to be minimum. This observation indicates that the bioflavonoids such as catechins and quercetin bound in binding pockets of the protein complex.

**Table 4:** Comparison of active hit compounds docked against modeled HCV NS3 GT3a.

Compounds	Molecular Formula	Interaction energy (kcal/mole)	HBonds
SID 17513061	C_16_H_11_NO_4_S	−85.165	1
SID 3716320	C_20_H_12_N_2_O_4_	−90.566	4
SID 17513201	C_17_H_14_N_2_O_2_S	−65.266	2
SID 17401675	C_14_H_14_N_4_O_2_	−110.633	5
SID 49666882	C_19_H_18_N_2_O_5_S_3_	−96.326	2
SID 24818609	C_11_H_11_BrN_2_O_3_	−89.623	1
SID 24827353	C_20_H_18_ClN_3_O_4_	−92.366	4
SID 49732586	C_21_H_16_BrNO_5_	−70.362	4
SID 4257236	C_18_H_21_N_5_	−94.238	6
SID 49817864	C_22_H_17_N_3_O_8_S	−89.365	3

## Perspectives on the Future Directions

This study as proved an impetus to initiate broader screening for key molecules as HCV helicase inhibitors. The lead compounds screened in this study should be a promising starter for large scale screening from the list of more than one million compounds from Ligand info meta database using the virtual screening protocols. Recent advances in understanding the molecular basis for helicase action might also spur interest in rationally designing compounds that should target key motif or clefts. HCV helicase is clearly an undeveloped drug target, which is envisaged with all the recent advances the pharmaceutical industry may track new attempts to find better helicase inhibitors. Such continued work and recent advances in the field will hopefully soon result in the discovery of more compounds for use in the laboratory and clinical trials. Deeper biochemical understanding of the complex nature of helicases will eventually help to define the molecular mechanisms of RNA helicases which will be a vital to attain an efficacious drug against human and animal pathogens and the ultimate assault on viral infections with their induced pathogenesis such as chronic liver disease. In summary we cloned, expressed, purified and docked GT3a NS3 helicase as a model with inhibitors. This study will pave a way to study in depth the molecular interactions of helicase of other HCV genotypes and help for screening specific antiviral inhibitors from combinatorial chemical library and random biological library.

## Supporting Information

Video S1 **Computational docking study with possible antiviral drugs and analysis of complex interactions.**
(MP4)
